# High Elevation Occurrences of Jaguars ( *Panthera onca* , Linnaeus 1758) (Mammalia, Felidae) in Northwestern Mexico, With a Record of Collaborative Interactions

**DOI:** 10.1002/ece3.72206

**Published:** 2025-09-25

**Authors:** M. Fernanda del Pozo‐López, Rugieri Juárez‐López, Carlos A. López González

**Affiliations:** ^1^ Facultad de Ciencias Naturales Universidad Autónoma de Querétaro Querétaro México

**Keywords:** camera trap, Chihuahua, coalition, habitat suitability, *Panthera onca*

## Abstract

The jaguar ( *Panthera onca* ) is an adaptable, solitary predator that used to range from Argentina to the southwestern United States. Despite having been extirpated in several areas due to anthropogenic activities, there is a breeding population in the State of Sonora, and resident adults in Arizona, in the United States. Even though the State of Chihuahua is in close proximity and there is a likelihood of dispersal, there are no formal studies for the species in the State. As part of a broader monitoring project, we surveyed two privately owned ranches and one ejido in the northern part of Chihuahua, Mexico, using camera trapping equipment. We obtained five new records of the species as well as one account of a potential collaborative interaction between two males. These records show the use of temperate environments and higher elevations, which contrast with published literature, as this species is commonly associated with tropical and sub‐tropical environments below 1200 m. We highlight the importance of the area and the need for it to be taken into consideration when developing models and conservation strategies.

## Graphical abstract

Jaguars in the northern end of their distribution have recently become the center of conservation efforts; however, the State of Chihuahua has been underrepresented in monitoring projects. We present five new records of jaguars in high altitudes as well as the first evidence of two jaguars exhibiting cooperative behavior in Mexico. We highlight the importance of considering the area when discussing conservation efforts.

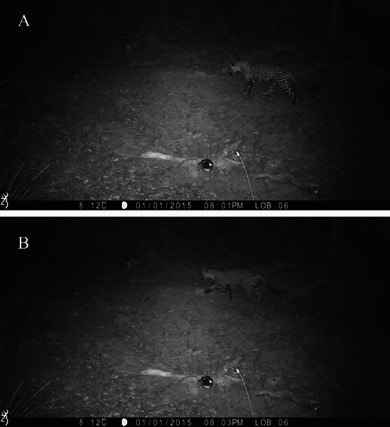

## 1 Introduction

The jaguar ( *Panthera onca* , Linnaeus 1758) is notably adaptable to a variety of environmental conditions and habitats (Seymour 1989; Hatten et al. 2005), which allowed the species to be present throughout the American continent. Its historical range once extended from the southwestern United States to northern Argentina. However, due to anthropogenic pressures, it was extirpated from several areas, constricting the species' range (Sanderson et al. 2002).

At the northern end of their current distribution, which includes the southwestern portion of the United States and the Mexican states of Sonora and Chihuahua, habitat associations represent Chihuahuan Desert scrub, pine‐oak woodlands, and tropical thornscrub, which reflect a transition from southern tropical vegetation habitats. Their presence in areas of montane oak and pine‐oak woodland is a phenomenon that contrasts with the typical lowland habits throughout the jaguar range (Van Devender et al. 2024).

The Mexican State of Sonora, where the northernmost breeding population is found (Brown and López González 2001; Boydston and Lopez Gonzalez 2005), together with Arizona and New Mexico, is a prime focus of conservation efforts in recent years with reports of occurrence (Rabinowitz 1999; Sanderson, Redford, et al. 2002; Sanderson, Fisher, et al. 2022). These efforts determined the establishment of critical habitat and the recovery planning process in the United States (USFWS 2010; Sanderson, Fisher, et al. 2022). In addition, these have resulted in various initiatives to recover the historic jaguar population (USFWS 2016, 2018; Sanderson and Fisher 2013). The State of Chihuahua, on the other hand, has remained hidden and not the center of attention, as there are no formal studies for the species in the State. Observations of jaguars in the State of Chihuahua were opportunistic and involved individuals killed for livestock depredation in temperate forests (Brown and López González 2001). Current knowledge shows few published reports of occurrence despite the spatial proximity to Sonora and the consequent likelihood of dispersal and an eventual recolonization into the southwestern United States. However, Chihuahua represents a considerable amount of potentially suitable habitat for northern jaguars, thus enhancing the importance and noteworthiness of the records we present here, not only because they are new occurrences for the area, but also because they may indicate that populations in the northwest have surplus individuals, which will in turn become colonizing adults. As a result, northwestern Chihuahua could become a fundamental part of the species recovery, aided by anthropogenic factors such as large‐extension ranching.

We aimed to document and analyze new jaguar records in Chihuahua, focusing on the significance of high‐elevation events for Mexico. We used recent literature and digital platforms to support our findings.

## 2 Methods

Jaguar records were collected as part of a broader wildlife monitoring project that included two privately owned ranches and one ejido (communal land) within the Municipalities of Janos and Casas Grandes, in the State of Chihuahua. One ranch and the ejido are located inside the Janos Biosphere Reserve. Vegetation types include grasslands/shrublands, oak woodlands, and conifer forests, and riparian vegetation. The area has an arid and temperate climate, with warm summers and winter rains, a mean annual temperature of 15.7°C, and a mean annual precipitation of 381 mm (CONANP 2013). The most prevalent economic activities for the region include livestock grazing and white‐tailed deer and turkey hunting concessions.

We collected wildlife data in four separate 30–35‐day period surveys intermittently between May 2020 and September 2023. In 2020, 12 Browning cameras model BTC‐5PXD and 10 model BTC‐5HDE were used. In 2021, the setup consisted of 5 Browning BTC‐5PXD, 3 Browning BTC‐5HDE, 2 WildView STC‐TGLX8IR, and 2 WildView STC‐WV30. In 2022, camera traps used included 6 Browning BTC‐5PXD, 9 Browning BTC‐5HDE, 3 Bushnell model 119736, and 12 WildView. Last of all, in 2023, the cameras used were 12 Browning BTC‐5PXD and 15 Browning BTC‐5HDE.

The sampling effort totaled 3131 trap‐days with 89 camera trap stations. Individual locality sampling effort for Casas Grandes (2020) was 805 trap‐days; Casas Grandes‐Janos (2021) was 475 trap‐days; Janos (2022) was 945 trap‐days; and lastly, Casas Grandes‐Janos (2023) equaled 906 trap‐days.

Camera traps were spaced between 1 and 2 km in abandoned roads, canyons, seasonal and perennial streams, depending on topography and accessibility. Stations were baited with sardine, oats, and corn with vanilla extract. We recorded coordinates and elevation data in the field using the Alpine Quest Off‐Road Explorer mobile app (AlpineQuest 2025) and hand‐held Garmin GPS devices. The equipment remained active 24 h per day and was set to take still pictures with a picture delay of 1 min and a multi‐shot mode of 3 shots, where 3 pictures were taken spaced 3 s apart. Memory cards were screened to be analyzed to construct and curate a database.

We identified jaguars due to their spot‐pattern coats with black spots in rosettes, yellow/orange fur, and large body size. The color of the fur and the rosette shape of the jaguars' markings are distinctive, becoming unmistakable characteristics for the individual identification of the species (Nelson and Goldman 1933). Photographs obtained by camera trap stations mentioned herein enabled accurate identification of the species. Side profiles of the individuals allowed us to differentiate them when comparable.

In order to understand the potential distribution of the species in Chihuahua, we searched for additional and previously published records (i.e., Brown and López González 2001), and those from the citizen science platform iNaturalist. Select records met the Research Grade category and had available visual confirmation of the species. The platform protects endangered animal observations, making them obscure, and thus the list of coordinates that can be downloaded is a random point within a 0.2 × 0.2‐degree block and consequently not precise (iNatHelp 2024). Hence, multiple occurrence points were merged into a single record to illustrate the presence in this given area. We also conducted a literature survey using multiple internet search engines with the keywords: jaguar; high elevation; and Mexico. We then performed a snowball protocol to obtain additional records. All data were plotted using QGIS v. 3.34.10 (QGIS 2023).

## 3 Results

The wildlife survey compiled the records of 25 mammalian species, representing 10 genera and four orders (Table 1). Carnivora was the order with the most species, followed by Rodentia and Lagomorpha. The localities of Janos and Casas Grandes–Janos had the highest value of richness, with 20 species recorded each. The latter, which was surveyed twice, once in 2021 and later in 2023, had an increase in value, growing from 17 to 20. Jaguars were recorded in all sites throughout the study, similarly to other felids, except for ocelots, which were only recorded once in 2023.

**TABLE 1:** List of mammal species and species richness per locality, result of the broader monitoring project to illustrate potential for the areas that were surveyed.

Order	Genera	Species	Casas Grandes	Casas Grandes—Janos	Janos	Casas Grandes—Janos
			2020	2021	2022	2023
Didelphimorphia	Didelphidae	*Didelphis virginiana*				X
Carnivora	Felidae	*Leopardus pardalis*				X
		*Lynx rufus*	X	X	X	X
		**Panthera onca**.	**X**	**X**	**X**	**X**
		*Puma concolor*	X	X	X	X
	Canidae	*Canis latrans*	X	X	X	X
		*Canis lupus baileyi*	X	X		
		*Urocyon cinereoargenteus*	X	X	X	X
	Ursidae	*Ursus americanus*	X	X	X	X
	Mephitidae	*Conepatus leuconotus*	X	X	X	X
		*Mephitis macroura*	X	X	X	X
		*Mephitis mephitis*	X	X	X	X
		*Spilogale gracillis*			X	X
	Procyonidae	*Bassariscus astutus*			X	
		*Nasua narica*	X	X	X	X
		*Procyon lotor*	X			
Artiodactyla	Tayassuidae	*Pecari tajacu*			X	X
	Cervidae	*Odocoileus virginianus*	X	X	X	X
Rodentia	Sciuridae	*Sciurus aberti*	X	X		X
		*Sciurus nayaritensis*	X	X	X	X
		*Neotamias dorsalis*	X	X	X	X
		*Otospermophilus variegatus*	X	X	X	X
Lagomorpha	Leporidae	*Lepus californicus*			X	
		*Sylvilagus audubonii*			X	
		*Sylvilagus floridanus*	X	X	X	X
		Species richness	18	17	20	20

*Note:* The highlight is point out the location of the species at all localities.

We obtained five new jaguar records at the three surveyed localities (Table 2). The oldest record was registered in the Casas Grandes Municipality, while the other four were recorded within the Janos Biosphere Reserve. These sites are located in vegetation types corresponding to coniferous forests, as they present a vegetation primarily composed of pine and oak trees that are commonly associated with higher altitudes.

**TABLE 2:** New records of jaguars in Chihuahua, Mexico, observations reported by the authors of this manuscript. Male (M), Indeterminate (I).

Date (d/m/y)	Sex	Municipality	Vegetation type	Elevation (m)	Latitude	Longitude
06/06/2020	I	Casas Grandes	Pine forest	2297	29.98075	−108.177072
28/12/2021	M	Casas Grandes‐Janos	Oak forest	2203	30.409227	−108.389239
21/07/2022	M	Janos	Oak forest	1752	31.23231	−108.766352
05/09/2023	I	Casas Grandes‐Janos	Oak forest	2227	30.421681	−108.400906
05/09/2023	I	Casas Grandes‐Janos	Oak forest	2227	30.421681	−108.400906

**FIGURE 1:**
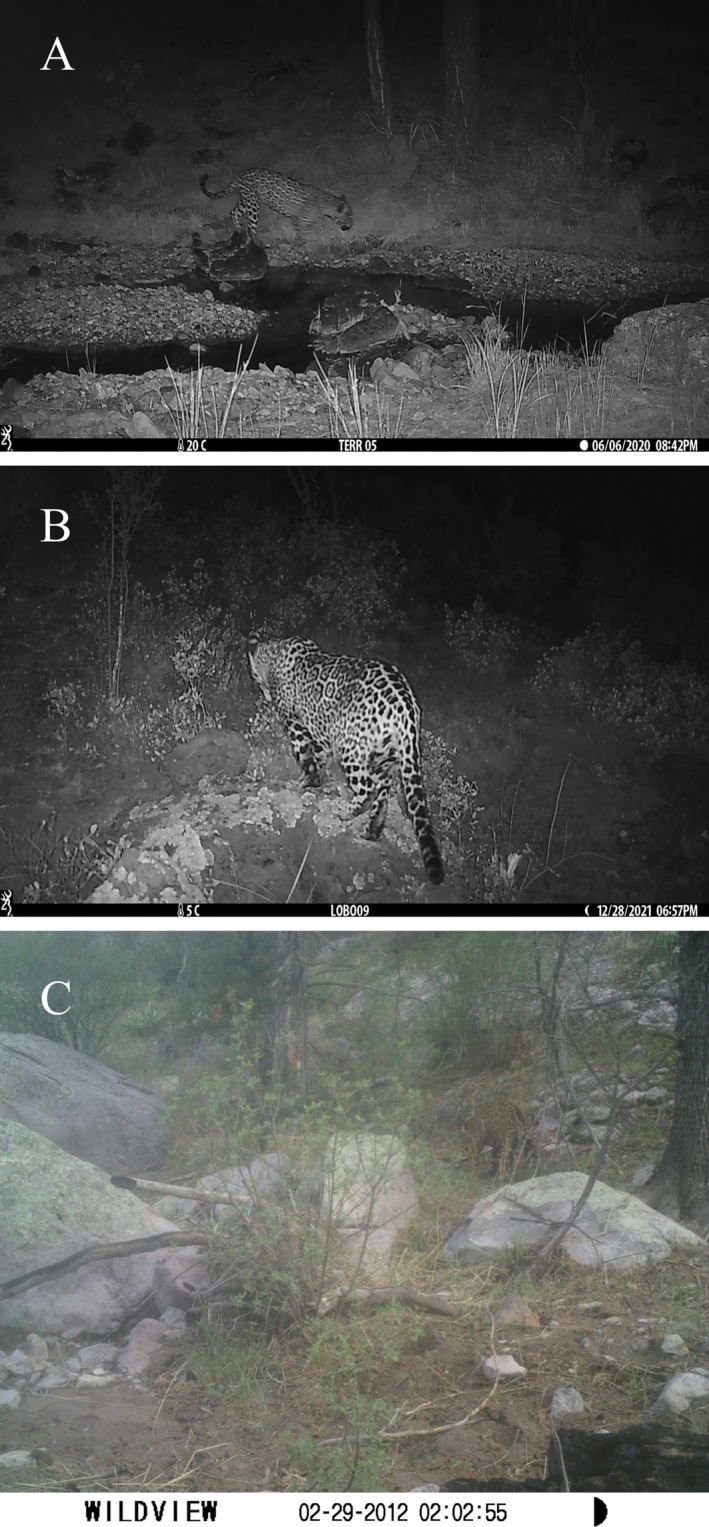
Camera trap snapshots of three of the new jaguar records presented. (A) Camera trap record from June 2020. (B) Jaguar record from December 2021. (C) Photograph taken in July 2022.

The first record (Figure 1A) was obtained on June 6th, 2020, at 2042 h, on a camera trap set close to the bank of a stream in the Casas Grandes location. This is a perennial water stream, as there is a spring a few meters uphill. Pine trees remain unexploited by the local owner to preserve the integrity of the body of water. Riparian vegetation is composed of pine forest, with surrounding oak and second‐growth vegetation such as manzanita shrubs (*Arctostaphylos* sp.), a result of wildfires that eliminated primary vegetation. There is no livestock in the area, but white‐tailed deer and wild turkey are managed as game species.

On the 28th of December, 2021, at 1856 h, there was another jaguar recorded (Figure 1B) on a camera trap that had been placed amidst a vegetation patch, with the closest road located 300 m south of the location point. This is a highly transited pathway because it is the only way to access nearby ranches in the Casas Grandes–Janos area. Vegetation is composed of pine‐oak forests, as well as second‐growth vegetation produced by wildfires. The area is used for extensive livestock grazing, and turkey and white‐tailed deer are harvested.

The third jaguar (Figure 1C) was documented on the camera trap that was set furthest from the group of cameras, closer to the mountain range, in an oak forest in the northeastern end of the Sierra Madre Occidental. Even though it was set higher in elevation than the rest of the cluster, it is the lowest altitude recorded for this set of jaguar records. The device was located in a dry, seasonal stream, although there is a livestock dam 2.3 km east of the camera location. Land is communal, and just like the other areas, livestock grazing is extensive, and domestic animals are moved between pastures depending on water availability. The camera, which was a WildView model STC‐TGLX8IR, reconfigured itself due to an electronic glitch or the likely rubbing of livestock. A picture was taken on July 21, 2022, at 07:01 h.

**FIGURE 2:**
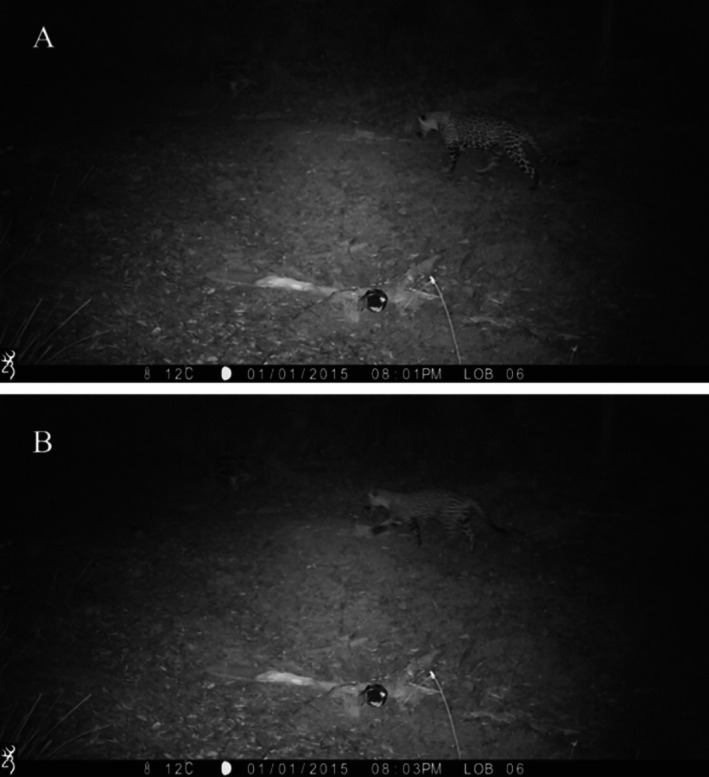
Camera trap records of the two different jaguars recorded in September 2023. (A) First jaguar, recorded in 2001 h. (B) Second jaguar, photograph taken in 2003 h.

The last two records, obtained on September 5, 2023, represent two jaguars. The detection events resulted in six photographs (Figure 2). The first three photographs show the first individual and were taken at 2001 h. The next three, taken with a 2‐min difference interval at 2003 h, represent a second individual who appears to be following the first one. Their relatedness remains undetermined, as does the sex of both individuals. However, historical records of the area have only reported the presence of males, with no females (Brown and López González 2001). Unfortunately, the Browning camera model BTC‐5HDE, which was set on an old road, was misconfigured when a cow impacted the device, but no information was compromised apart from the date, which returned to the default setting.

There are 19 records on the iNaturalist platform that appear to be associated with temperate forests. The majority of these were from 2020, 2021, and 2022, with one record from 2017 and another from 2018. This data were distributed throughout the year with no discernible pattern. The distance from this region to the northernmost breeding population in Sonora is 67 km. In addition, we compiled nine jaguar observations from recent literature between 2010 and 2025 that were found above 2000 m. These sightings were all of males and included individuals from the states of Durango, Zacatecas, Jalisco, San Luis Potosí, and Oaxaca.

## 4 Discussion

**FIGURE 3:**
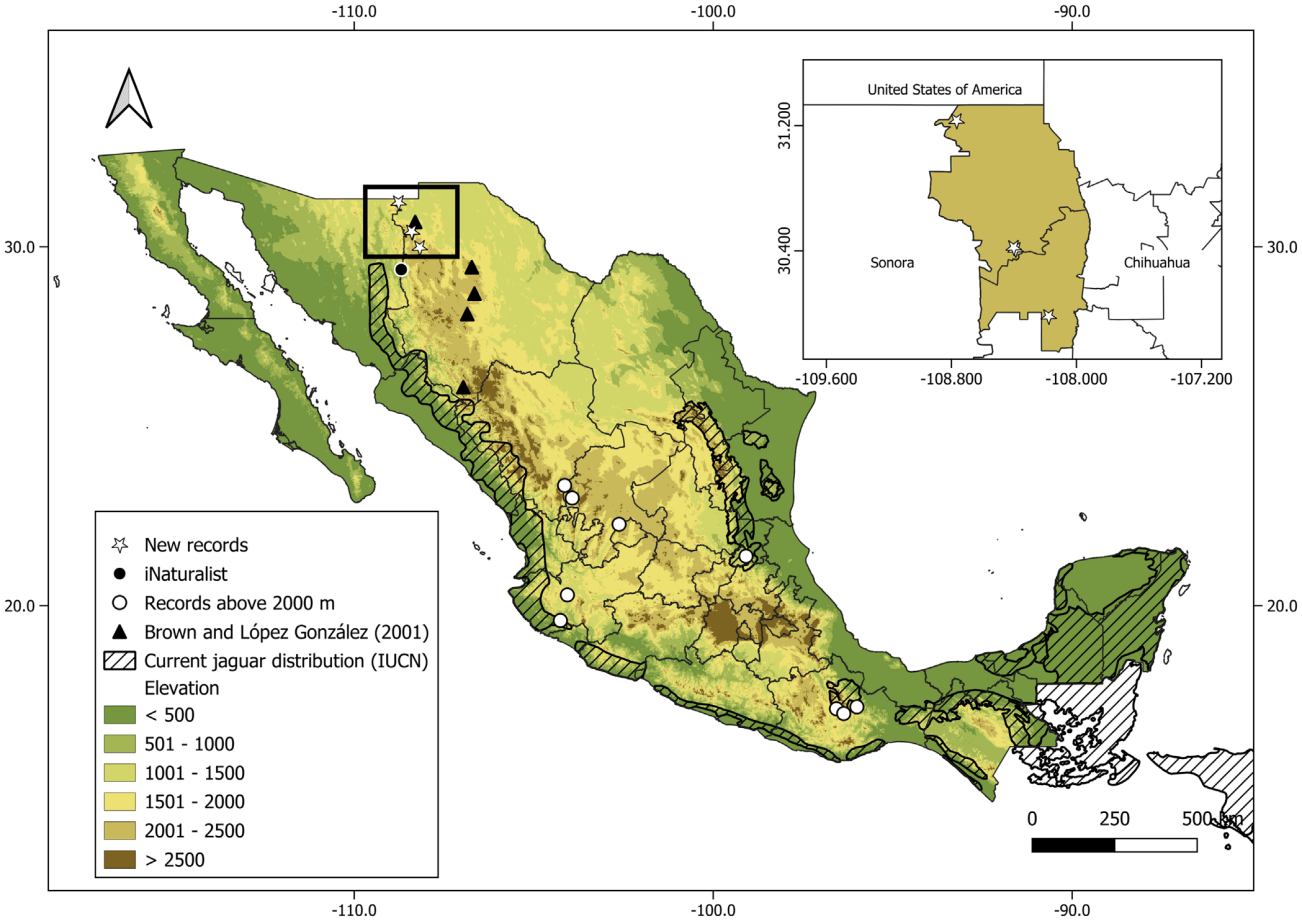
Jaguar records for Chihuahua and high elevation in Mexico. Data sources for the map are new records, Chihuahua published reference (Brown and López González 2001); iNaturalist (2025), clustered as a single point; high elevation records (sources Villordo‐Galván et al. 2010; Aranda et al. 2012; Briones‐Salas et al. 2012; Padilla‐Gómez et al. 2019; Rodolfo Pineda Pérez CONANP pers. comm. 2025; Moreno‐Arzate et al. 2022; Quintero‐Díaz et al. 2024).

These new records from Northwestern Mexico are noteworthy because they correspond to an underrepresented area within the historical range. Brown and López González's (2001) pioneer efforts to compile verified records of jaguars in the northwestern portion of their range through data of animals reportedly killed in Chihuahua between the years 1957 and 2000 clearly show an association with mountainous terrain for Sonora and Arizona (López‐González and Brown 2002; McCain and Childs 2008). Our records show an association with temperate environments and higher elevations, contrary to most literature, as this species is strongly associated with tropical and sub‐tropical environments below 1200 m (Sunquist and Sunquist 2002; Quigley et al. 2017; Van Devender et al. 2024), despite extraordinary reports of jaguars at altitudes above 3000 m (Trujillo et al. 2025). However, similar habitat associations have been registered in central Mexico, highlighting jaguar presence in pine‐oak forests above 1800 m (Monroy‐Vilchis et al. 2009). Comparably, the Gran Sierra Plegada in San Luis Potosí, which is part of the Sierra Madre Oriental, documented a pine‐oak forest record as well as a high‐elevation photo taken at 2400 m (Villordo‐Galván et al. 2010). Current records of jaguars in mountainous environments may represent habitat refuges from prosecution, rather than high‐quality habitat and an array of potential of available prey (Villordo‐Galván et al. 2010). There are currently few reports of Mexican jaguars in habitats above 2000 m (Figure 3), and in the rest of their distribution, less than 1% of the records occur above this mark (Trujillo et al. 2025), which highlights the relevance of the records presented herein in the context of the country and the northernmost areas of jaguar distribution.

Because all jaguars were male, they were thought to be transients from east‐central Sonora. However, these males, which were thought to be young, could have been looking for a mate or aiming to establish a territory for themselves, as this species had been thought capable of inhabiting the southwestern portion of the State (USFWS 2018). The latter could be the case of the jaguars here reported. The jaguar recorded in December 2021 (Table 2) was documented on March 13, 2023, in the Northern Jaguar Reserve (NJR) in Sonora, where the northernmost known jaguar population occurs (USFWS 2018; Carmina Gutiérrez‐González, pers. comm. 2024). This male traveled over 120 km from its initial sighting in Chihuahua (Table 2) to the NJR in Sonora. Additionally, there is a report of a male jaguar that left the Reserve and subsequently returned (Van Devender et al. 2024).

Currently, there are no female jaguar reports in the study area. Suitable habitat for males has been identified for the State of Chihuahua (Boydston and Lopez Gonzalez 2005; Torres‐Olave et al. 2023). This responds to their ability to survive in areas where reproductive females are restricted. This takes place for the most part near reproductive populations and low‐quality habitats (Conde et al. 2010).

Northwestern Chihuahua has long been excluded from analyses of distribution and possible connectivity areas for jaguars (Rodríguez‐Soto et al. 2011, 2013; de la Torre et al. 2018). However, the same area has been identified as high‐quality habitat for large carnivores such as Mexican gray wolves ( *Canis lupus baileyi* ), American black bears ( *Ursus americanus* ), and pumas ( *Puma concolor* ) (González‐Saucedo et al. 2021). Moreover, a critical connectivity corridor has been recognized in the Mexico–USA border, allowing dispersal for the previously mentioned species. In the USA portion, jaguar sightings have been reported in southwestern Arizona in the last three decades (Babb et al. 2021). Sanderson et al. (2022) highlight that the Arizona–New Mexico states currently have potential habitat suitable for the species' recovery. The aforementioned area is located north of our records, which emphasizes the importance of Chihuahua as a potential recolonization pathway for jaguars.

Over 50% of the animals in this survey are potential prey for jaguars (Table 1). Large mammals such as white‐tailed deer ( *Odocoileus virginianus* ) and collared peccary ( *Pecari tajacu* ), which were recorded in the surveyed areas, as well as medium‐sized species, including white‐nosed coati ( *Nasua narica* ), which were present in all three surveyed sites, are a favored prey species throughout the jaguar range (Núñez et al. 2000; Villordo‐Galván et al. 2010; Rueda et al. 2013; Cassaigne et al. 2016; Ávila‐Nájera et al. 2018). Other medium‐sized species such as raccoons ( *Procyon lotor* ), gray fox ( *Urocyon cinereoargenteus* ), skunks ( *Mephitis macroura* ), opossum ( *Didelphis virginiana* ), and lagomorphs have also been recorded through dietary analysis, although less frequently (Ávila‐Nájera et al. 2018; Cassaigne et al. 2016; Rueda et al. 2013). Acknowledging a substantial prey base and diversity, the northern part of the State of Chihuahua becomes a relevant area, with potential not only as a stepping‐stone but also as a suitable permanent jaguar habitat.

Other efforts of monitoring projects include the Flora and Fauna Protection Area Campo Verde, located on the border between Sonora and Chihuahua. Their jaguar photographs, a result of camera trapping surveys, were uploaded to iNaturalist (2025) in 2020, 2021, and 2022. The area is over 100 km away from the new records presented here, but gives insight into the presence of the species.

In this regard, points of occurrence from Campo Verde, Janos, and Casas Grandes should be taken into consideration in the future when developing models and conservation strategies, as the current distribution consensus (Quigley et al. 2017) and priority areas have not considered the possible occurrence of jaguar in Chihuahua (Rodríguez‐Soto et al. 2011). Moreover, jaguar‐specific projects and studies are needed to better comprehend the population, which, despite being made up in its entirety of males, can still provide valuable information for the species.

For instance, the last two records, which depict two jaguars that appear to be traveling together, illustrate what could be considered a potential collaborative behavior. Since both records were taken by the same camera trap within 3 min, with a temporal proximity that indicates that they were within sighting distance, the interaction could be hypothesized as possible cooperation or coalition according to Jędrzejewski et al. (2022), given that they were allegedly following each other. Alternatively, multiple jaguar presence occurs at sites with surplus carrion available (Guilder et al. 2015). This record is spatially limited to one camera trap station; however, it is to our knowledge the first evidence of what could be considered cooperative behavior in Mexico. More information is needed if these jaguars are transient males or if they have become resident, as has happened in Arizona (McCain and Childs 2008), and to ascertain any relationship.

These accounts add to the current understanding of Mexico's jaguars, especially those on the northern end of the distribution. More studies are needed in regard to population and interactions in the State of Chihuahua, in order to obtain a better scope not only of the current areas of distribution but also of the social life of these solitary felids. These new records do not represent isolated events but rather an extension throughout a geographical strip over 170 km on the northeastern Sierra Madre Occidental. Furthermore, the results presented here contribute to the knowledge of the species distribution in this region, which is not necessarily taken into consideration when discussing jaguar‐related matters due to a generalized lack of information. Our data could be used to better model the species range and the partially and completely occupied areas, as well as more precise corridor areas of dispersal between other parts of Mexico and the USA.

## Author Contributions

**M. Fernanda del Pozo‐López:** conceptualization (equal), data curation (equal), formal analysis (equal), investigation (equal), methodology (equal), validation (equal), writing – original draft (equal), writing – review and editing (equal). **Rugieri Juárez‐López:** conceptualization (equal), data curation (equal), formal analysis (equal), methodology (equal), supervision (equal), validation (equal), writing – original draft (equal), writing – review and editing (equal). **Carlos A. López González:** conceptualization (equal), formal analysis (equal), funding acquisition (equal), investigation (equal), methodology (equal), supervision (equal), writing – original draft (equal), writing – review and editing (equal).

## Ethics Statement

The authors have nothing to report.

## Conflicts of Interest

The authors declare no conflicts of interest.

## Data Availability

All relevant data is within the manuscript.
